# Stakeholder perspectives on implementing geographic information systems for leprosy elimination in Pakistan: A qualitative study

**DOI:** 10.1371/journal.pone.0360428

**Published:** 2026-11-05

**Authors:** Vincent Geskus, Anil Fastenau, Rhea Brüggemann, Fabian Schlumberger, Johan van Soest, Matthew Willis

**Affiliations:** 1 Department of Health, Ethics and Society, Faculty of Health, Medicine and Life Sciences, Maastricht University, Maastricht, The Netherlands; 2 Marie Adelaide Leprosy Center (MALC), Karachi, Pakistan; 3 Department of Global Health, Institute of Public Health and Nursing Research, University of Bremen, Bremen, Germany; 4 German Leprosy and Tuberculosis Relief Association (DAHW), Wuerzburg, Germany; 5 Rawalpindi Leprosy hospital, Rawalpindi, Pakistan; 6 Brightlands Institute for Smart Society (BISS), Faculty of Science and Engineering (FSE), Maastricht University, The Netherlands; 7 Department of Radiation Oncology (Maastro), GROW Research Institute for Oncology and Reproduction, Maastricht University Medical Centre+, Maastricht, The Netherlands; Para Federal University, BRAZIL

## Abstract

**Introduction:**

Leprosy remains a pressing global health concern, with Pakistan among countries still facing missed and delayed diagnosis. By highlighting elevated risk areas, Geographic Information Systems (GIS) are a cost-effective solution to improve leprosy case detection by guiding targeted interventions to high-risk areas. This study’s purpose was to investigate the perceived GIS implementation facilitators and barriers, to support targeted interventions and advance the Zero Leprosy strategy.

**Methods:**

Semi-structured interviews were conducted with twelve stakeholders, including local field staff, program managers, and international experts. Thematic analysis was guided by the Unified Theory of Acceptance and Use of Technology (UTAUT) and the Diffusion of Innovation Theory (DOI) to identify facilitators and barriers concerned with GIS implementation for leprosy.

**Results:**

Key facilitators included an integrated multi-disease and pilot-based rollout approach, capacity building activities, offline-capable tools, and improved visibility to drive motivation. Barriers included the limited local capacity in complex GIS data analysis, hybrid digital-manual workflows, historical data lacking GPS, and ensuring feedback and ownership for staff members.

**Conclusion:**

GIS implementation is not only dependent on individual readiness, but also on broader external and organizational facilitators and constraints. This necessitates holistic implementation strategies that recognize the interconnectedness of different parts of the system. Internal capacity building is paramount to address statistical complexity and to build trust, ownership, and independence. As the world moves closer to achieving Zero Leprosy, integrating GIS within a multi-disease approach will be critical to making sustainable progress in combatting neglected tropical diseases.

## Introduction

Leprosy, a highly stigmatised neglected tropical disease (NTD), still persists as a pressing global health concern with over 200,000 new cases annually [1,2]. Early diagnosis and prompt treatment are critical to prevent disabilities and halt further transmission [2]; however, underreporting exacerbates disease control challenges, making detection of missed cases a priority [3]. While crucial for the detection of new cases, traditional active case detection (ACD) activities can be resource-intensive and difficult to scale, especially in areas and countries with financial or logistical restrictions. WHO and the Global Partnership for Zero Leprosy (GPZL) promote digital innovations to achieve rapid and sustained progress towards complete elimination [4].

One such innovative approach is the use of Geographical Information Systems (GIS). To support ACD, spatial analysis techniques like GIS are seen as a promising digital tool to inform control strategies, through their ability to highlight regions of high endemicity for targeted interventions [5]. It enables the analysis of variables that play important roles in disease transmission [6,7].

Compared to conventional methods, GIS offers a cost-effective approach to leprosy control by identifying hotspots and directing resources to high-need areas [6,8]. In Brazil and Indonesia, heatmaps identified clusters where future cases were likely to occur, demonstrating GIS’s potential to improve outcomes of ACD efforts and reduce costs [9,10]. Next to cost-effectiveness, GIS can assist with the choice of ACD activities, based on the spatial analysis of epidemiological indicators over time. Because leprosy clusters at various geographic levels, from households to broader community areas, GIS cluster analysis techniques can guide the most effective strategies for each area, which study results demonstrated [6,11]. For example, in Bihar, India, hamlet-level analysis revealed high-risk clusters beyond the household level that would benefit from active screening and exposure-related preventive treatment [5].

In Pakistan GIS could support more targeted ACD. While considered a low-endemic country since 1996, Pakistan reports ~300 leprosy cases annually. The presence of visual deformities and child cases is a concern as it reflects diagnostic delay and ongoing spread [12]. The continued presence of leprosy in Pakistan coexists with surveillance gaps, lack of coordination and limited funding [13,14]. These are relevant factors for the adoption of geographical tools to enhance coordination and support targeted intervention to overcome diagnostic delays [15,16]. Although some initial implementations are already taking place, GIS in Pakistan has yet to be fully implemented. Typically, GIS adoption remains limited due to high implementation costs, unskilled workforces, limited access to GIS infrastructure, data availability and privacy issues [17,18].

This study aims to explore the contextual challenges and opportunities associated with implementing GIS mapping for leprosy, applying a qualitative approach with the research question: *‘’What are the barriers and facilitators in implementing GIS mapping for efficient active case detection in Pakistan from the perspective of healthcare workers and key stakeholders?”* The research is expected to offer insights particularly relevant to low-resource settings, contributing to the targets of Zero Leprosy.

## Methodology

### Study design

This study employed a qualitative research design, involving semi-structured interviews with stakeholders relevant to the leprosy context in Pakistan, associated with the Marie Adelaide Leprosy Centre (MALC) and Aid to Leprosy Patients (ALP). This approach was chosen for its flexibility to provide rich insights on the ongoing implementation of GIS and other digital health solutions, and the related challenges and opportunities, from a professional perspective. Topics covered range from implementation challenges, and technical issues to sociocultural factors and expected benefits. Due to travel restrictions the interviews were conducted online between April 14 and May 9, 2025.

### Study Setting & Population

This study focused on the whole country of Pakistan, with specific attention to Karachi, the country’s largest city with over 20 million inhabitants. Pakistan is classified as low-endemic, but the Sindh province, with Karachi as its capital, faces the largest portion of new cases [19]. Pakistan has no official national leprosy control programme. Instead, two civil society Non-Governmental Organizations (NGOs), MALC and ALP perform most of the country's leprosy activities [13].

GIS pilots in Pakistan ran parallel to this study. Fig 1 shows a simplified timeline of GIS implementation in Pakistan for leprosy control to date. Implementation at ALP was less widespread and limited mainly to a few initial introductions. Work is currently underway to introduce and integrate a DHIS2 system for GIS [16]. Implementation is expected to start in 2025 and should eventually be rolled out nationwide (Fig 1).

**Fig 1:**
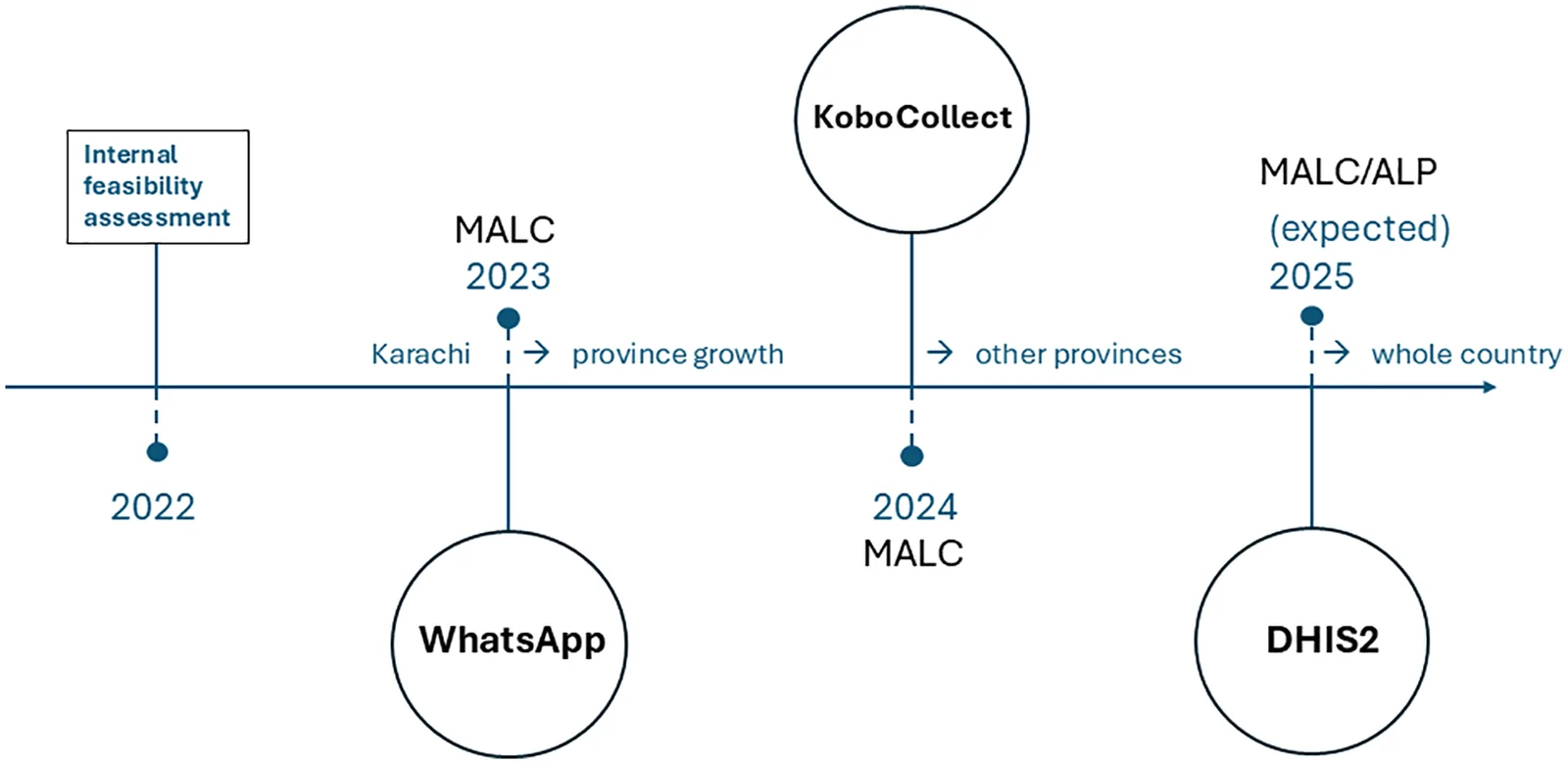
Timeline GIS in Pakistan.

This study used a purposeful sampling plan. Invitations were sent out to *leprosy health professionals* with experience in the Pakistani context, who were involved in some way in the use or management of GIS. This categorization refers to various kinds of healthcare workers (e.g., nurses and medical doctors), as well as to managers, field officers, and data analysers. This study also included *international experts* in the field of leprosy and/or GIS to provide a broader perspective and incorporate global insights, improving generalizability.

In total, this study included twelve participants: six international experts and six local leprosy professionals. Out of the twelve participants, there were eight men and four women participating. The average participant age was 46 (24–70 range). Recurring patterns across participants indicated that key themes were likely well captured, and that thematic saturation was reached.

### Data Collection & Theoretical Framework

This study focuses on the *people* component of GIS—those who use, manage, and support the system—while recognizing the role of technical and organizational infrastructure (Fig 2) [20]. 12 semi-structured interviews of approximately 60 minutes were conducted in English via Zoom. An interview guide (S1 Appendix) has been used to direct conversations towards predeveloped themes using standardized questions. The questionnaire was prepared in advance based on prior literature as well as the Unified Theory of Acceptance and Use of Technology (UTAUT) and the Diffusion of Innovation (DOI) frameworks of technology implementation [21–23]. It was evaluated by two researchers in the field of leprosy and adjusted accordingly. After the first interviews, the guide was revised again to address identified gaps.

**Fig 2:**
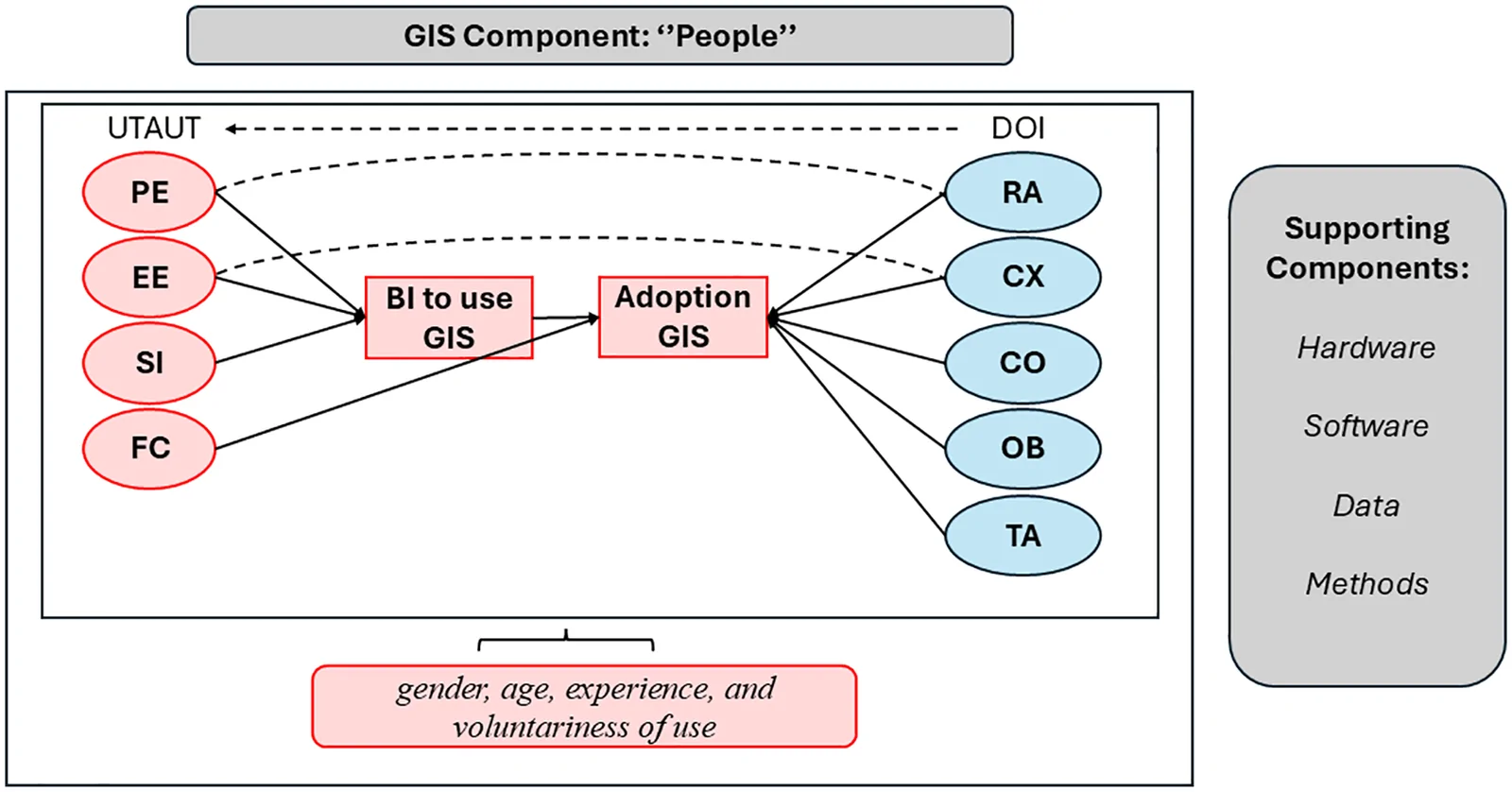
Conceptual model UTAUT/DOI.

Drawing on UTAUT, this study examines how performance expectancy (PE), effort expectancy (EE), social influence (SI), and facilitating conditions (FC) shape behavioural intention and actual use [21]. Complementing this, DOI offers insights into broader contextual and cultural dimensions, highlighting relative advantage (RA), compatibility (CO), complexity (CX), trialability (TA), and observability (OB) as key determinants of innovation diffusion [22]. Merging the domains of UTAUT and DOI provides a comprehensive framework (Fig 2) to identify the facilitators and barriers influencing GIS acceptance and use among leprosy professionals in Pakistan. Combining the frameworks adds practical advantages, as for example, compared to the UTAUT, the DOI’s compatibility (CO) element allows the consideration of more cultural components, which may specifically be important in a country like Pakistan. Accordingly, the interview themes centred around the participants’ background, their views regarding the value of GIS in Pakistan, and their implementation recommendations. The thematic domains of the UTAUT/DOI framework were also used to analyse, structure and display the results.

### Data Analysis

The interviews were analysed using Naeem et al.’s six-step method for thematic analysis [24]. After transcribing the recordings verbatim, data were analysed through an iterative process of identifying and organizing keywords, codes and themes; a process that continued until no new patterns occurred. The data was grouped into seven categories based on the UTAUT and DOI frameworks, and displayed by identifying key facilitators and barriers, in the results section. These elements contributed to the development of a new conceptual model, providing a well-structured interpretation of the findings [24].

Quotations were used in a table divided by the four subpopulations (1. International experts, 2. MALC office staff, 3. MALC field staff, 4. ALP office staff) to illustrate typical findings or variations in perception on each facilitator and barrier through the respondent’s own voices [25]. Quotations were presented anonymously, containing only the interview number, and were rewritten in case of severe grammatical and language errors. Stutters and filling words were left out.

### Ethical Considerations

Ethical approval was obtained from the Medical Ethics Review Committee (METC) of Maastricht University (*FHML/HDT/2025.024*) and the MALC Ethics Committee (*MAC/ERC/01*). Participation in this study was completely voluntary and written informed consent was obtained from all participants prior to involvement. The participant information sheet and consent forms are attached as S2 Appendix and S3 Appendix respectively. Participants kept the right to withdraw from the study up until anonymization of their data. All interviews were recorded, transcribed and stored without any personal information and will be deleted ten years upon study completion to guarantee data privacy and confidentiality.

## Results

### Performance Expectancy and Relative Advantage

Participants across all subpopulations expressed optimism about GIS’ potential to improve the efficiency and effectiveness of ACD activities, optimize resource allocations, and disease control in Pakistan. This belief emerged as a key driver among the local staff to adopt GIS. Many expressed that it would ease their work in the long run and will prove valuable to reach Zero Leprosy, especially since there was a common belief that the traditional control methods have reached their limits.

*“I am very positive … we can do our future planning, minimize our resources and use these at the right place. This is very positive”* [8]

This perception was influenced by the low-endemic status of leprosy in Pakistan, which complicates planning of control activities amidst declining budgets. In this light, GIS is seen as a “final push” to eliminate leprosy, given its ability to provide targeted insights to guide field workers to potentially endemic areas.

An expressed concern was however about over-relying on GIS as a strategy, especially if it drifts attention away from other, more vital processes, like ensuring the availability of high-skilled dermatologists in Pakistan. Additionally, international experts stated that the current GIS approach in Pakistan can be improved by including new innovative techniques like genomic surveillance. More common social phenomena within Pakistan, particularly the high mobility of the population due to internal labour migration, were viewed by some local participants to potentially undermine the reliability of GIS data.

*“Karachi is a mobile shifted population … so we also have this issue, as sometimes patients are not found because they live somewhere else”* [10]

Not accounting for such movements, for example by adding multiple locations per registered patient, was believed to limit GIS’s utility in understanding disease spread, leading to scepticism about the effectiveness of GIS under such conditions. However, as pointed out by international experts, migration of patients would be a problem regardless of GIS. Another expert expressed uncertainty about the technical feasibility of adding multiple addresses for internal migration.

### Effort expectancy and Complexity

Although respondents believed that GIS will eventually improve efficiency and reduce workload, many expect the opposite during early implementation. Key concerns were: time needed to learn to use the system (incl. field staff and data analysers), time spent collecting data (on paper and digitally, and collecting GPS locations for legacy data) and processing all data accordingly. A participant noted that this will create such a workload increase, that it might create resistance for this person and their co-workers, especially without compensation – which was not expected to be provided. Others, while recognizing the increase in workload, did not regard it a major issue, considering it part of their job.

*‘’they have to keep the manual record, and they have to do it digitally. So, the work is doubled …* [8]

While participants anticipated few issues on the GIS data collection front, regarding it as relatively easy to learn, drawing meaningful conclusions from the data was considered a more complex task. This was attributed to low levels of knowledge in basic epidemiology and data analysis skills, with only one person at the organization equipped with some basic knowledge.

*… if people are not able to interact with GIS, people may resist to adopt … it is a natural thing that you must resist against that thing”* [4]

A local participant expressed optimism about this and believed that they would gradually learn the more complex parts over time. However, an international expert noted, although they may not yet realize it themselves, that the complexity of GIS analysis could create resistance among those expected to learn it, as it is difficult to learn, requiring significant training. Other complexities were to translate the knowledge gained from data analysis into plain language, so that decision-makers and lay people can understand it. One expert suggested to appoint and educate a dedicated *focal point* who can communicate technical outcomes to non-technical stakeholders.

Local participants’ perception of GIS’s complexity was moderate, whereby most considered it relatively easy to learn the aspects required for their function – considering it not to be so-called “rocket science” – given that appropriate training was provided. For instance, two local field workers collecting digital GPS data for over two years noted that they now consider this task simple, but they made many errors (e.g., wrong data entries and duplication of efforts due to difficulties in navigating the system) in the beginning due to limited training. It was expected that the new DHIS2 tool would re-introduce some complexity, requiring new training to prevent such early-adoption errors.

All participants constantly emphasized such importance of extra training, not only to build an adequate skill set, but also to build confidence and reduce the perceived complexity of the tool. According to the participants, trainings should thus go beyond mere technical instructions (e.g., collecting and analysing geographic data), including attempts to address fears (e.g., fear of human replacement by technology) and to build trust (e.g., believing that you can learn it) to overcome potential local resistance, especially among senior staff members. They would be more reluctant to change, and training was seen as an important tool to transition away from old working routines. Multiple experts noted that such training should be layered and tailored to specific roles to avoid one-size-fits-all expectations and to support the ease of use.

*‘’I think step one of these trainings is showing what the added value is. And then once they are motivated, then for the technical aspects, that's something you can learn. ‘’* [11]

Participants also recommended recurrent sessions to create a strong internal capacity, to support ongoing training and scaling up through so-called “training of trainers”, to reduce long-term dependency. Both local and international participants believed that the local workforce is strong enough to absorb the needed extra knowledge, skills and mindset.

### Social influence

Participants noted that longstanding efforts in leprosy control of the involved organizations, and the strong reputation they have built in this time within the communities, contribute positively to the acceptance of new technologies and innovations among community members. This includes, but is not limited to, GIS. Several local participants expected that this position will help to ease any concerns among patients and their direct surroundings, concerning data collection, care delivery, privacy, and stigmatization. The feeling was that, as long as they believe that the new ways of doing improve the care that MALC and ALP are able to deliver, there will be a sense of trust and no strong resistance.

*“MALC is a very high appreciated organization in Pakistan … people in rural areas see a car with a sign saying MALC and were always happy. And so, I think there was almost no resistance towards any technology”* [12]

However, some participants did express concerns about potential resistance (e.g., as this was an issue in other countries), necessitating the need for specific and tailored awareness campaigns to empower community members.

Several local participants described how there is a sense of enthusiasm among their colleagues and upper management for using the GIS systems, which had a positive effect on their own motivation and confidence. Also, influential figures, both internal (e.g., managerial) and external (e.g., academia) partners, who advocate for GIS within the organization were seen as a guiding force in taking up this innovation by creating a sense of urgency. One local person noted that such “respected figures” are important to convince the workforce of the overarching strategy and the role GIS can play in the entire leprosy project, as their views on the matter were trusted.

*“They [influential figures] are guiding us for using these digital things … They know the whole project, which is obviously beneficial for leprosy. So, it will be beneficial for us too.”* [7]

However, the results showed varying organizational cultures which influence the adoption of GIS among individuals, which the experts stressed need to be anticipated.

### Facilitating conditions

Internet within certain geographical areas in Pakistan was considered unstable. Digital data collection tools that were dependent on internet connectivity (i.e., the early WhatsApp tool), had led to frustration among field workers, who experienced repeatedly that locations could not be captured or uploaded to the server. This led to lost data and left them discouraged as work had to be re-done. It even got to the point where one field worker used to consider unreliable internet as the main barrier to GIS data collection.

*‘’… you should definitely not rely on internet while collecting data”* [2]

International experts recognized that data collection should not be reliant on connectivity, which led them to design and introduce applications that can log GPS locations offline and upload it when connection is restored (i.e., Kobo and future DHIS2).

International help, especially in the initial years of setting up and implementing GIS, was deemed essential for success by the participants. These (mainly western) partners were described as important to bring in funding, troubleshooting, for creating local capacity by providing trainings through their expert knowledge on GIS, and building motivation and trust among the workforce.

*“… If they do not support us, we are not able to do this GIS … after implementation we still need support from GLRA … to continue training and support for things like hardware and software, we cannot do this without them.”* [10]

The help was perceived as consistent and reliable, but some feared for the sustainability of the leprosy projects if funding or other support were to be withdrawn, pointing towards dependency. Some even saw this support as a legitimizing force to the GIS project, as they would not do it independently. Experts expressed that their support is planned for the first three years of the project, while gradually transferring capacity to local staff to reduce dependency. However, some local participants indicated that there should remain continuous support after the initial implementation, to provide training and technical support.

No hardware issues were expected by the participants. Regarding the hardware needed to collect GPS points, most participants suggested that the field staff can use their own mobile devices. Pakistan was perceived as “digitally ready” and mobile phones were seen as an integrated part of society, resulting in widespread device ownership, which would make this a pragmatic, cost-effective and to the staff familiar approach to collect data.

*“I use my smartphone and this tablet … most in my team are very young and they are very prone to use these smartphones”* [7]

One expert noted from evidence abroad, that allowing the usage of personal phones had better outcomes than providing dedicated devices. Local participants also considered this a user-friendly approach, especially for the younger people. However, additional work tablets were deemed beneficial to overcome some limitations, such as staff members who do not possess an up-to-date phone and issues that were inherent to smartphones (e.g., small screen and limited battery life). Also, it was said that compensation (e.g., internet packages) must be provided for. One ALP participant feared however that personal data could be at stake when installing the GIS software on their phone, fuelling resistance among coworkers to use their own device.

Most participants did not witness or expect potential legal challenges to GIS implementation. They even painted a positive picture concerning government involvement, or perhaps better said: the lack of government involvement. Legal institutes were said not to be too involved, which created a dynamic where there is a lot of freedom in program implementation. In the Sindh province, the government even provided an explicit “no objection certificate”.

*“… we have the permission … but we do not know in other provinces, we get the permission normally or not … so we have to find out solutions.”* [8]

However, others highlighted the need for caution in other Pakistani regions, where political institutes or military forces would not grant permission. For example, one expert explained that staff pointed out that certain areas are very strict and that it is illegal to collect GPS information in the field.

### Compatibility

The leprosy control efforts in Pakistan over the past decades were praised by the international experts, contributing to the country’s low leprosy rates. However, now with the goal of Zero Leprosy, there was a broad agreement among the stakeholders that it is not sustainable to use GIS solely for leprosy control. Continuing only on leprosy was seen as impossible, as less work would be available and funding would decline, making GIS for just leprosy a dead end.

*“If this experience is relevant for leprosy, then it can be translated to cutaneous leishmaniasis … to integrate for sustainability”* [1]

Integrating other skin diseases, such as cutaneous leishmaniasis or scabies, was suggested for sustaining GIS initiatives beyond leprosy, to retain knowledge and expertise gained, and to ensure relevance for the trained personnel. Various respondents pointed out that such a holistic way of working is compatible with current workflows, as the responsible organizations already work across disciplines, including tuberculosis (TB) and blindness control, which made them feel positive towards such a strategy.

There was a general resistance among local staff to fully part from traditional, manual data collection, expressing the wish to collect data both digitally and on paper. This hybrid approach was seen as a way to ensure reliability and ownership of the data and was ultimately seen as more compatible with their values, even if it would take more time. Even in the longer term, several participants did not expect to stop collecting data on paper.

*“We have to keep everything on paper. I think that once it will be maybe only a small card for them”* [8]

While not rejecting digital data collection, there were some trust issues (e.g., fear of system failure and technical reliability in case of natural disaster), while manual work was considered a preferred and safe addition. International experts recognized these beliefs among local staff but were convinced that such beliefs would fade over time when they build trust. A hybrid approach was therefore overall seen as a valid strategy to give the staff time to smoothly transition away from traditional methods, and to learn to gradually trust the reliability of the digital records; however, it was mentioned that this does need to be phased out over time to avoid inefficiencies (i.e., duplication of effort) and data quality issues by lack of consistency.

While the historically collected data, all of which has been digitized, were generally praised for their consistency, standardization, and high quality, leading most participants to believe that there will be few integration issues, some participants did point out a limitation with this data. They pointed out that this data does not contain GPS coordinates, nor was it based on a functional and consistent address system, which was said to make them (partially) incompatible with the GIS software.

*‘’it did not work to connect the address information to any GPS location within GIS because there was no consistency in how the addresses were stored … there is no real working address system in Pakistan”* [12]

Mainly locally, there was a belief that it would require them to re-visit all historical locations to gather GPS information, which was considered a difficult and time consuming task, especially as there are nearly 60,000 registered patients, which adds to the workload. A process which was said to be made even more complicated by the mobile population of Pakistan. International experts did not believe there is such a need to re-visit all of the old locations, all the way back to 1980s. They stressed that they could just simply make a good estimate by using the centroid of a village from before a certain date, even if this would limit the usefulness of that data for targeted interventions.

### Observability

GIS’s output, especially the creation of visuals and maps, was seen as a stimulating tool to enhance motivation within the workforce and stimulate behavioural change. For example, one international expert expected that from the moment the data analysers are motivated and skilled, it will work “contagiously” as the “fancy” reports will start spreading throughout the layers of the organization.

*‘’If we talk about leprosy, I think it is very important to show them success. At the same time, success also means that there is less need for leprosy control activities”* [2]

Another expert noted that in another country, pride and engagement increased when a geo-based mapping tool was implemented and results were clearly visualized. At the same time, data integrity improved as accountability increased. Next to motivating the workforce, some mentioned it could also help convince stakeholders and funders by providing extra depth when writing proposals. Local participants expected that GIS will provide them with more insight into their work, which will ultimately create a positive depiction of their work by visualizing their efforts and improve their work performance. Additionally, it was said that it could bring a sense of joy in their work by creating a play-like dynamic for finding new cases.

Introducing a GIS dashboard, providing tailored information per function in the health system (incl. access control to ensure data privacy), was seen as a useful tool to directly show the GIS results.

A local participant indicated that they currently experience a sense of disconnection from the data they collect once submitted through the digital collection platform, as they lose the access to and thereby the ownership over their data. It was discussed how only the statistics department has the permission to retrieve and analyse this data, while the field staff must wait for a monthly report. This lack of real-time insight into the results was said to lead to a feeling of disempowerment and exclusion, which further reinforced the idea of continued paper records usage as a method to retain access over their collected patient records. To overcome this issue, the staff member mentioned that they would benefit from acquiring direct access to the data, albeit in a limited form. Various experts coined the idea of integrating a dashboard, which ideally also works on the smartphone of the field staff, that can create automatic reports to mitigate this issue.

### Trial-ability

Local participants stressed that there was a feeling that there was the possibility to experiment with innovations, like GIS, on a limited basis. Feedback was said to be taken seriously, and changes were being made based on their suggestions. This perception of an organizational willingness to engage in iterative learning cycles created a safe feeling that they could first ‘try it out’ before moving on to large scale implementation. This was described as an important aspect to build confidence and a positive attitude towards the innovation. As another local participant explained, initially, when they started with digital data collection, they considered the required data collection form too extensive. It was not possible to fill it out in one go during field visits, which led to a feeling of discontent and more errors. After providing feedback, the collection form was altered to be more practical and user-friendly.

*‘’So, we were trying, we were doing mistakes all the time, and it was very long. We discussed it… and now he made it very short … so it is now very easy for us”* [7]

This aligned with advice from an international expert, that stressed that GIS tools should be created through a phased and co-created approach, instead of top-down.

International experts noted the importance of such a gradual, small-scale implementation strategy, as it allows for adjustments to the technology (e.g., adjusting variables to local context and taking out system failures) and the implementation strategy over time. Current regionalized efforts were generally seen as an ideal way to learn and adjust, before implementing such systems all over Pakistan.

## Discussion

The qualitative approach of this study identified facilitators and barriers to implementing GIS for leprosy control in the Pakistani context from a multi-stakeholder approach. Individual roles, experiences and organizational context all appeared to influence stakeholder perception, highlighting the importance of considering multi-stakeholder perceptions in implementation efforts. The local staff revealed a general willingness and intentions to use GIS; a positive sign for real-life usage behaviour [21]. While some potential barriers arose regarding the workload and compatibility with existing practices, such as a desire to stick to paper-based data collection, these did not appear to undermine GIS adoption intention. The international experts foresaw few implementation issues, mainly requiring internal capacity to be built to address data analysis challenges to reduce external dependency. Although this study demonstrated differences in perceptions between international experts and local staff, the research population was too small to draw definite conclusions about these differences.

The study highlights a multi-faceted framework influencing GIS adoption for leprosy in Pakistan, involving external, organizational and individual factors (Fig 3). The model may provide guidance in different locations after careful consideration of context-specific individual, organizational, and external factors. The first, external layer captures the broader, environmental context influencing the adoption. The second, organizational layer reflects the internal cultural dynamics, including the ideas on how compatible GIS is with current and old practices. The third, individual layer represents personal skills and beliefs influencing GIS adoption. The complex and multi-layered dynamics necessitate holistic implementation strategies: strategies that do not think in siloes but instead recognize the interconnectedness of different parts of the system.

**Fig 3:**
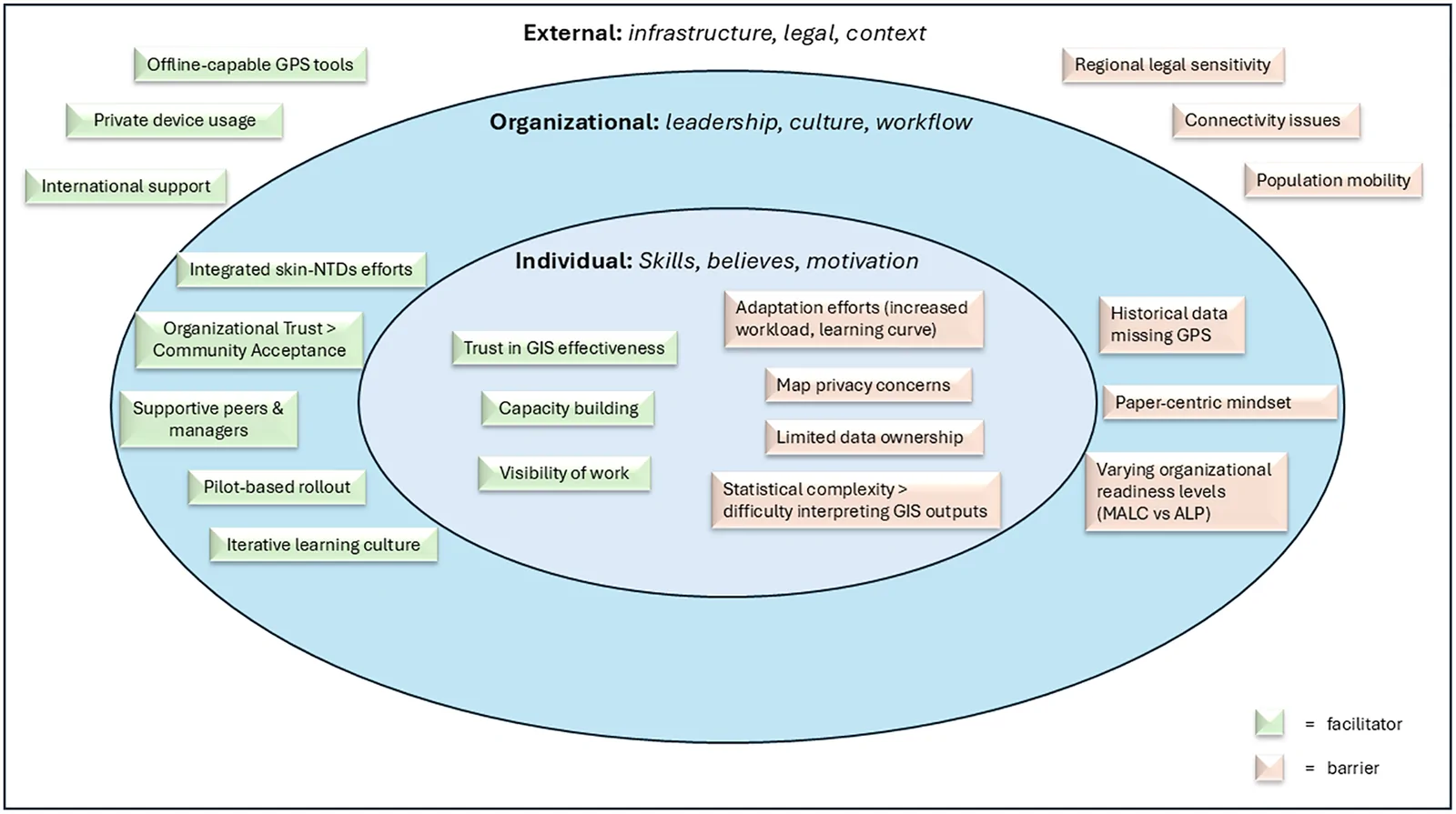
Conceptual Model of Multi-Faceted Framework Influencing GIS Adoption in Pakistan (Source: Author’s own).

Existing literature reported how a triad of factors was among the greatest challenges to implementing GIS for public health purposes: 1) Limited local technical capacity (e.g., improperly trained staff capable of GIS related activities), with heavy reliance on external technical assistance, 2) Limited availability of data, and 3) Lack of infrastructure, including limited access to expensive GIS software [18]. These challenges are exacerbated in low-resource settings like Pakistan [26]. Especially the first point was echoed in this study, considering the stakeholder’s perception of complexity in GIS data analysis and the highlighted importance of the international NGO and academic partners, which were deemed important for providing technical assistance and were even seen as the legitimizing force behind the implementation process. This study however, by incorporating individual readiness components, also explicitly emphasizes the importance of addressing mindset and motivation to empower and create a sense of trust as part of effective training.

Considering the second factor, this study shows that data issues are also apparent in Pakistan, despite the availability of digitally stored data on leprosy cases spanning over multiple decades (incl. around 60,000 patients), as a lack of a standardized address system and thus a lack of GPS locations renders the data (partially) incompatible with GIS. It requires vast amounts of work to make this data useful for spatial analysis purposes, for example by re-visiting the old locations. Research indicates that good modelling and planning of epidemics requires extensive and diverse datasets, which are said to be complicated to acquire, especially in developing countries [7]. This suggests that the lack of GPS in historical leprosy data could be a challenge to implementing GIS in Pakistan. Yet, the actual implications remain unclear and cannot be determined from this study, requiring additional investigation.

Lastly, while infrastructural challenges were less prominent in this study, concerns around internet connectivity and private device usage suggest that these challenges are also relevant to the Pakistani context, albeit they are a bit more subtly expressed. Also, given the partial implementation of GIS tools in Pakistan, some infrastructural challenges like internet issues were overcome by the introduction of a freely available, open-source offline tool, i.e., Kobo Collect. This importance of offline tools in GIS adoption, overcoming user issues, overlaps with other research. Fisher & Myers argue that, to truly democratize the power of health GIS, non-internet tools need to be available to the developing countries, as there is a continuous struggle with internet access [26]. Dependence on web-based GIS applications will in comparison tend to re-centralize the power of GIS to the privileged who can rely on continuous and reliable access to internet [26]. This implies that not only data collection should be available offline, as often highlighted in this study, but so should the software to perform GIS analysis to allow usage at (rural) district centres.

In contrast to the literature, privacy and confidentiality concerns appeared less present in this study [27]. Research indicates that balancing the need for detailed spatial information with the protection of individuals is a difficult task [28]. Stakeholder concerns remained limited to some slight privacy concerns related to the mapping procedure. However, there is a need for transparent data collection (e.g., asking informed consent) to prevent stigmatization [29], yet it is unclear how local cultural dynamics in Pakistan experience this and how it affects the broader acceptance of GIS. Community perception is however just one part of the concept of privacy. On the more technical side of data privacy, safeguards were put forward as solutions and mitigation to privacy concerns, such as limiting the use of patient data to anonymous, aggregated syntheses. While aggregated mapping is indeed seen as a valid strategy to de-identify geographic information [30], it is still important to be aware of privacy issues within other parts of the GIS process. Further literature stresses the importance on including modules on confidentiality and stigma considerations in GIS capacity-building activities [30].

### Limitations

In comparison to prior research, this study explored a wide range of implementation factors, not remaining exclusive to technical challenges, but also considering factors of personal readiness. Furthermore, by involving a wide range of stakeholders this study gained insights into a broad spectrum of perspectives. This enabled a nuanced understanding of the challenges and opportunities for GIS from a multi-stakeholder view, aligning with the study’s explorative objectives. However, due to the qualitative nature of the approach, the findings are not fully generalizable. Future research is needed to create more comprehensive long-term evaluations of GIS effectiveness, feasibility, and acceptance.

Interviews were conducted while the implementation process of GIS, albeit in limited form, was already underway. While the final GIS set-up intended for full-scale implementation, the so-called DHIS2, is yet to be finalized and therefore thus not used in the Pakistani context, some of the participants did already gain some hands-on experience. While conducting interviews mid-implementation can offer valuable real-time insights, it also has its limitations, as it neither provides a clear pre- or post-implementation view, nor collects continuous data over an extended period of time. This might have compromised the depth of this study’s findings. Also, the partial rollout of GIS in Pakistan might have impacted the perception between MALC and ALP stakeholders, whereby various levels of familiarity created different perceptions. Given the cross-cultural aspects of this research, the research process and interpretation were inevitably influenced by the researcher’s positionality, especially given that the primary researcher conducted interviews remotely. An extended positionality statement is attached as S4 Appendix.

### Recommendations

Finally, several practical recommendations emerged from the findings that can be applied to the observed ongoing project, as well as cautiously adopted in a varying context (Table 1). Especially, cross-cutting aspects can be found in existing literature, such as the need for capacity building, phased change management and privacy concerns. Empowerment through training leads to increased ownership of data collection and analysis with less external support necessary [18,27,31]. Clearly communicated change, nevertheless, should be undertaken through a gradual phasing out [26,32–34].

**Table 1:** Recommendations for GIS Implementation.

Recommendation	Core Focus	Key Insight
1. Train Local Health Workers in GIS Tools	Building local capacity and ownership.	• Provide practical and role-specific training, with recurrent follow-up; including both technical GIS skills (*analysing geospatial data; focussed and standardized procedures of parameter selection, data displaying, cluster analysis and data interpretation*) and social skills and attitudes (*mindset, fears and motivation).* • For field staff training, use familiar devices (own phone) with open-source software. • Appoint a *focal point* (one or two dedicated persons) that translates GIS findings into plain language for policy creation. • Empowering local staff to take ownership over digital shift could reduce dependency on international support in the long run to secure continuous use.
2. Expand the Integration of GIS Beyond Leprosy	Multi-disease strategy.	• Consider integration with other NTDs (*e.g., cutaneous leishmaniasis, scabies*), and other diseases such as TB, to support integrated, multi-disease intervention strategies. • Would show a sustainable way forward, optimizing resource allocation and ensuring programme sustainability. • Such an approach supports the retention of (technical) GIS capacity beyond leprosy elimination, continued funding and shows staff a sustainable path forward.
3. Increase Visibility through Real-Time Data Access	Data feedback and motivation.	• Timely access empowers (health) staff to track their performance, perform peer comparison, and show their distance-to-targets. • Introduce dashboard functionalities for frequent, real-time data visualizations (including mobile function). • Consider digital literacy skills and align dashboard interface; provide appropriate training where necessary.
4. Temporarily Respect the Co-existence of Old Practices and Habits	Change management.	• Allow a gradual shift from paper-based to digital methods, instead of an abrupt change. • Clearly communicate the necessity for change (e.g., by explaining why hybrid methods are sub-optimal, as it can increase errors and time spent). • Actively involve staff from the beginning in the planning and phasing out the old working methods to create psychological ownership and reduce resistance.
5. Consider Regional Differences	Tailored implementation strategies.	• Recognize regional and organizational differences across the country of implementation. • Use pilot-based rollout to build trust and confidence among users, facilitating a gradual shift. • Adapt locally tailored approach to support a unified national GIS strategy.
6. Explore Innovative GIS Applications	Future innovation and research.	• Explore the integration of GIS with innovative approaches such as *genomic surveillance* and *Geo-AI.* • Consider data, privacy, ethical and workforce challenges for appropriate use of such innovations. • Strengthening workforce capacity in advanced epidemiology and spatial analysis for general GIS usage remains essential for effective use of such innovations – and should be prioritized before (widespread) implementation of new applications.

## Conclusion

GIS is a transformative tool for public health, and an essential part in the global fight against leprosy and other NTDs. This research found a wide variety of factors influencing the adoption of GIS for leprosy control in Pakistan, with individual, organizational and external components playing an interconnected role. With the GIS-DHIS2 application on its way, the main challenge remains to build a strong local capacity, not only to address the lack of statistical skills and experience, but also to create a sense of trust and confidence among the workforce and to reduce the dependency on international support. As the world moves closer to the goal of eliminating leprosy, integrating GIS mapping and spatial analysis; both within leprosy control and through a broader multi-disease approach, will be critical to making sustainable progress in combatting NTDs. Ultimately, this will contribute to the road to Zero Leprosy.

## Supporting information

S1 Appendix Final Interview guide. (DOCX)

S2 Appendix Information sheet. (DOCX)

S3 Appendix Informed Consent. (DOCX)

S4 Appendix Extended positionality statement. (DOCX)

## Abbreviations

ACD: Active Case Detection
ALP: Aid to Leprosy Patients
BI: Behavioural Intention
CO: Compatibility
CX: Complexity
DOI: Diffusion of Innovation Theory
EE: Effort Expectancy
FC: Facilitating Conditions
GIS: Geographic Information System
GPS: Global Positioning System
MALC: Marie Adelaide Leprosy Centre
NGO: Non-Governmental Organization
OB: Observability
PE: Performance Expectancy
RA: Relative Advantage
SI: Social Influence
TB: Tuberculosis
UB: Usage Behaviour
UTAUT: Unified Theory of Acceptance and Use of Technology
NTD: Neglected Tropical Disease; WHO, World Health Organization
