# Supplementary material for: Stakeholder perspectives on implementing geographic information systems for leprosy elimination in Pakistan: A qualitative study
Source: PLoS One. 2026 Nov 5;21(11):e0360428. doi: 10.1371/journal.pone.0360428 (PMC13739172; doi:10.1371/journal.pone.0360428)
Supplement: Supplementary file 1 [file pone.0360428.s001.docx]

## Appendix 1: Final Interview Guide

**Warming up (take around 5 to 10 minutes to get people familiar and comfortable + check if the purpose off the study and their rights are understood by all participants)**

- How are you feeling today? Did you have a cup of coffee this morning? How has the weather been lately?
- Have you ever participated in an interview study completed by a student / on this topic before? Is the purpose of this study clear to you? Do you have any question before we get started?

**Intro – Background Information**

- Can you tell me briefly a bit about yourself and who you are?
  - ***Age, gender, profession, and years of experience with leprosy (in Pakistan) – explain that is information will not be used in an identifiable way***
- Can you briefly describe your role in the healthcare/leprosy sector?
  - How did you begin working with leprosy and those affected by the disease
  - How many years of experience do you have in the healthcare/leprosy sector?

**UTAUT**

Performance Expectancy

- How **familiar** are you with GIS tools and its application in public health?
  **Give additional information if necessary**
  - What is your opinion about them? Do you foresee any **implementation issues? Or what eases the implementation?** *(this serves as a more ‘general’ question at the start of the interview, to identify the participant’s initial thoughts, without being influences by the other questions in the interview)*
- Do you think GIS will **improve outcomes** in leprosy control in Pakistan compared to current practices? Outcomes can be:
  - *Disease control / improved and earlier case detection*
  - *Cost efficiency / optimizing resource allocation*
  - *Time savings / earlier detection*
  - *Improved motivation for work / job satisfaction*
- In your experience, how often is health data used to inform decisions – do you think GIS output would really impact/change leprosy control efforts in Pakistan?
- Do you think that using GIS can **improve equity** in leprosy control by reducing (geographic/racial) disparities? **OR** do you think that using GIS can decrease equity, for example by increasing **stigmatization** in targeted communities?
- Is **patient migration** considered within GIS / how do you believe this impacts GIS quality?

Effort Expectancy

- Do you believe that you / LPs have enough **experience/expertise** related to using digital technologies at work (*so not just GIS tools*)?
  - What **other tools/software** are you/other people familiar with?
  - Is there a **general trust** among LPs in using digital systems / do you trust it?
- How difficult/easy do you think it will be for you / LPs to **learn and use** GIS?
  - What **challenges** do you think will arise for involved LPs when using GIS – considering internet access, hardware availability and digital literacy?
  - What kind of **training or support** would be necessary?
  - What **(design) factors** would make GIS more intuitive and user friendly?
- How do you think GIS affects your **workload** or that of other LPs?
  - Would it save time or add additional tasks?
  - Can it have a **negative emotional load** for LPs? – *e.g. they are overwhelmed or intimidated by system complexity / too many digital tools which requires change*
- Do **female LPs** face different barriers to using/accessing digital technologies like GIS?
- Do you think there are enough trained people in Pakistan with expertise in GIS and disease mapping / is the **workforce capacity** strong enough?
  - What could be done to strengthen this capacity?

Social Influence

- In what way could **influential figures** influence GIS adoption among Pakistani LPs?
  - Influential figures can, among others, be:
    - *Political figures*
    - *Religious leaders*
    - *General celebrities (e.g. famous sports players)*
    - *NGOs/MALC managers/directors OR other local (health) authorities*
  - Are there strong GIS **champions** in the organization (e.g. key decision-makers or leaders)? What difference would it make if someone like that supports it?
- Do you feel that decision-makers/leaders are actively advocating GIS adoption?
- How would you describe the attitude in MALC to new technologies/interventions?
  - Does GIS feel like a high priority within your organisation?
- Considering previous digital health tools, have you experienced any **adoption resistance** among LPs – can you explain what and wherefor this was?
  - If so, how was delt with such resistance within the organization? Do you feel that this was delt with adequately?
  - Did such hesitancy influence other LPs in their adoption decision – do you think that the **influence of colleagues** is important in the decision to adopt GIS?
  - Can you imagine **people fearing losing their job** with new innovation/GIS?
- Do you think that there might be **patients/communities resisting** the usage of GIS? Reasons for resistance could be, among other things:
  - *Data privacy / Fear of stigmatization*
  - *Considering GIS / digital health incompatible with traditional medicine*
  - *General distrust in leprosy organization*
  - Do you think patients will trust MALC for using this tool?

Facilitating Conditions

- Do you have the necessary **knowledge/resources** to use GIS for leprosy control?
- Is the current technical infrastructure—such as **availability of mobile phones, tablets, and reliable internet**—sufficient to support GIS-based data collection and usage?
  - What **technical challenges** *(e.g. internet connectivity, device malfunction, power outages)* do you anticipate?
  - Would you / LPs need **new** tablets/**phones**? Would you resist to use your device?
  - When technical problems arise, is there **support available** — and how effective is that support in your experience?
  - Do these challenges differ between **different areas within Karachi**?
- Do you think the **current (healthcare) policies, infrastructure and governance** in Pakistan supports the use of digital tools / GIS?
  - Are there any **legal, bureaucratic or political barriers** that you foresee?
    - *Privacy rules, unclear/ambiguous rules, etc.*
  - Do you believe there is **sufficient funding** to support GIS?
  - How does the **lack of a national leprosy programme** affect implementation?
  - In your opinion, do the relevant **governmental stakeholders** play a positive or negative role in GIS implementation? (*e.g. gov. targets/incentives)*
  - How do you see the role/important are **international, NGO, academic partners (cosmopolitanism)**? What if there support falls away?
  - In your opinion, **who should be involved** in the GIS implementation decision making process and **how do you envision their roles**? *Is it clear, now GIS gets introduced in Pakistan, who does what (data entry, analysis, decision-making)?*
- What would be needed to ensure **long term sustainability** after implementation of GIS?
  - What risks do you foresee (e.g. when initial, international implementation help halts)? What resources (funding, human capital, knowledge, etc.) are needed?
  - Who should be responsible to ensure consistent use? *(team, person, department)*
- What are your thoughts on the **availability, quality and reliability** of data in Pakistan?
  - For example, are these available / reliable:
    - *leprosy or spatial data, such as detailed maps, facility, GPS locations*
  - Do you believe that you / LPs have the **skills to input data** in a high quality way? (*complexity, effort expectancy*)
- How do you think the **costs and/or licensing restrictions** for GIS software impact the implementation and upscaling in Pakistan? 🡪 role of QGIS

**DOI**

Relative advantage – N/A

Compatibility

- Does GIS fit well into the **current workflow**? Do you think you can **learn GIS, while keeping current work going**; or would this take too much time away from other work?
- Can you shortly **explain the current digital systems** that are being used in Pakistan/leprosy control (e.g. EHR, data reporting systems, etc.)?
- Do you believe that GIS aligns well with the Pakistan (healthcare) infrastructure?
  - Are there any **integration issues** you foresee?
    - *Inconsistent/outdated geo-data (e.g. areas without GPS coordinates)*
    - *Poor system interoperability (e.g. EHR/DHIS2 system compatibility)*
    - *IT infrastructure/internet access*
  - Can you think of any technical or institutional issues that might come up when trying to link GIS to existing tools like DHIS2 or EHRs?
- Do you think that LPs and the (digital) systems across Pakistan use **consistent naming conventions and standards** when reporting data?
  - If not, how does this impact disease mapping efforts like GIS?
  - Are there any **policies to support the standardization** of data sharing?
  - **How important is it to follow standards for data sharing** and system integration – and do you think such standards are currently followed in Pakistan?
- What **cultural/societal factors** potentially influence the adoption of digital health tools in Pakistan? And how does this relate to GIS?
  - Are there **cultural beliefs/misconceptions** about technology, leprosy, or others, which you believe that might influence the acceptance of GIS?
- Do you have any **privacy/ethical concerns**, given the sensitivity of the data, to use GIS tools (low confidentiality)?
  - Have you seen any efforts to protect privacy or sensitive (health) data used in geographic mapping systems – do they work or not?

Complexity – *N/A*

Observability

- How important is it that LPs see the direct (positive) consequences of their work? And how does this relate to GIS – do/need they to notice what the benefits are?
  - If you are not informed about how your efforts (or delayed), would this affect motivation/trust in the system? How do you normally get informed?
  - Have you experienced irritation if you collect data, but this is not acted upon?
- Is there a challenges in measuring immediate results/impact from GIS implementation? To what extent has your organization/unit set goals for implementing GIS?
  - Will feedback from staff be collected/do you feel like your feedback matters?

Trial-ability

- How important is it (for you) to test GIS on a small scale before scaling up?
  - What is a good way to test GIS in Pakistan? What are success measures?

**Enablers and Barriers**

- Conclusion: what are to you the **main barriers**/**enablers** to implement GIS in Pakistan?
- Have you ever seen a new tool introduced that initially seemed promising, but failed to be adopted/scaled up? What happened, and why do you think it didn’t succeed?
- **Behavioural Intention**: do you intend to use GIS for leprosy control in Pakistan?

**ASK FOR RECOMMENDATION!**

**Ending**

- Is there anything you want to add? Is there a topic we did not touch, but which important to you for the successful implementation of GIS/digital technologies in Pakistan?
  - What questions would you ask other respondents *(project manager, statistic department, field officer, medical director, field worker, and leprosy/GIS experts)?*
- Thank you for your participation! Would you like to reflect/comment on the interview? Would you like to receive an update on the results of the study when these are available?
