# Supplementary material for: Stakeholder perspectives on implementing geographic information systems for leprosy elimination in Pakistan: A qualitative study
Source: PLoS One. 2026 Nov 5;21(11):e0360428. doi: 10.1371/journal.pone.0360428 (PMC13739172; doi:10.1371/journal.pone.0360428)
Supplement: Supplementary file 2 [file pone.0360428.s002.docx]

Appendix 2: Information Sheet

**Title of the Research**: Mapping the Neglected, Making them Visible; *Qualitative Study Investigating the Role of GIS Tools and Spatial Analysis to Tackle Leprosy in Pakistan*

**Institutions**: Marie Adelaide Leprosy Centre (MALC) Pakistan; University of Maastricht (UM)

**Principle Investigator:** Vincent Geskus, Student Health and Digital Transformation (UM)*;*
**Co-Investigators:** Anil Fastenau (MALC); Johan van Soest (UM); Matthew Willis (MALC),

Dear Participant,

Thank you for considering being part of this study. I am Vincent Geskus, a Dutch Master student Health and Digital Transformation at Maastricht University in the Netherlands. This research will be conducted for completing my master thesis and will be used to write a scientific paper that shall be published in a Global Health journal, like PLOS One or PLOS Global Public Health.

Please read this information sheet carefully before deciding to give informed consent and agree upon participation in this study. All information should be listed on this sheet, but if questions, doubts or concerns remains, do not hesitate to contact me directly (see contact information below).

**Participation Information:** You are being asked to take part in a study being performed by Vincent Geskus, on the instructions of Marie Adelaide Leprosy Centre (MALC) in Pakistan. You will participate in a one-on-one interview, which will be held online. This interview can be held in English or in presence of a translator. Before providing additional information about the nature of this study, it is important that you understand the following general principles that apply to all participants in this study:

1) Participation is entirely voluntarily at all times.
2) You are entitled to withdraw from the study at any given time without providing an explanation, without this impacting your reputation with MALC or any other harm.
3) If left with any questions after reading this information letter, we highly encourage you to seek contact using the contact information provided in this letter. We will gladly further inform you to understand the study more clearly.

**Introduction to the Topic:** You are being asked to part to take part in a study which seeks to investigate the challenges and opportunities for implementing Geographic Information Systems (GIS) to support active case findings for leprosy. Every year, there are still over 200 newly detected cases of leprosy in Pakistan. Leprosy, if left untreated, can lead to serious impairments and it allows the disease to infect new cases. The disease is curable with multidrug therapy. Therefore, it is highly important to detect new cases as early as possible. GIS tools can assist in early case detection, by highlighting areas of high risks. In response, policies can effectively be targeted on areas which need most help. This does not only result in better detection of new cases but is also proven to be cost-effective. To support the implementation of GIS tools for leprosy case detection in Pakistan, this study will perform interviews with relevant leprosy stakeholders in the area of Karachi. The goal of these interviews will be to identify real-world barriers and enablers, providing a comprehensive understanding of the feasibility and adaptability of digital health solutions in Pakistan, like GIS. These insights will ultimately form actionable recommendations, to support implementation efforts of digital health solutions in developing countries.

**Who can participate in the study:** Upon receiving this information letter, you can consider yourself eligible to participate in this study. You have been chosen based on your connection to MALC, being considered a relevant leprosy professional.

**Procedures** Upon approval to take part in this study by filling in the informed consent form, you will be asked to take part in a one-on-one interview, through a protected ZOOM-link. An interview will last between 30-60 minutes. This interview will be audio-recorded on an external recording device. In addition to the audio recordings, the researcher will take additional notes using pen and paper during the interview. These interviews will be conducted in English where possible. Where not possible, a translator will be present, to act as an intermediate between the researcher and you as a participant.

**Risks:** While limited to 30-60 minutes, participation will require a time-investment on your part, which might inflict with your professional responsibilities. Discussing challenges related to leprosy control might invoke distress when sensitive topics are raised. At such times, you will remain able to not provide an answer, without providing a reason for this. Appropriate safeguards minimize risk related to privacy and unauthorized access to your data.

**Benefits**: While there is no direct reimbursement, your participation will contribute to relevant research for leprosy control in Pakistan. This might ultimately improve your professional outcomes, given the aim of this research to contribute to the implementation of digital health tools in developing countries through creating actionable insights.

**Data Management:** All data collected throughout the research process will be handled carefully and stored safely. The consent forms, the notes taking during the research, and the interview recordings and transcripts will be stored by the main researcher, within password-secured files on a laptop. Research data will be stored for a period of 10 years following the last publication, in line with publishing guidelines. Data will not be shared with any external parties outside MALC and UM. MALC will retain the right to reuse the data from interviews in further research in the future. In this case, your data will be used under the same conditions as in this research, meaning you keep the same safeguards and rights.

**Confidentiality:** The interviews will be recorded on an external recording device. The responses in this interview will be analysed anonymously, and any personal information irrelevant to this study will be deleted at point of analyses. You can receive the transcript to review and suggest any necessary changes. In case of future publication of this study, data will be presented in a non-traceable way. Some personal data can be used to provide extra depth into the analysis, such as age, gender and type of professional. Such information will however only be used on an aggregate basis, to prevent information from being linked back to individuals so that no one will be able to identify you from study reports.

**Ethical Approval:** This study is in line with ethical guidelines. To conduct the interviews, ethical approval has been obtained from both the ethics committee of Maastricht University and from MALC . This approval has been granted based on an extensive list of ethical considerations and safeguards to which this study answers.

**Voluntariness**: Participation will at all point remain entirely voluntary, retaining the possibility to withdraw from the study at any time, without providing any reason. Once the data has been analysed, the anonymized data insights cannot be removed anymore. Although your actual data file will still be deleted. Also, you will remain the right to skip questions in the interview if you wish to do so. This will not impact your personal or professional relationship with MALC or UM and neither will this results in a penalty or damage in any other way. If you would like to withdraw from the study, please contact X at (TELEPHONE NUMBER +XXXXXXXXX).

**Contact information**; If you have any questions about the research at any time or if you feel that you have been harmed by taking part in this study, you can contact X at (TELEPHONE NUMBER +XXXXXXXXXX) or contact the principal researcher, Vincent Geskus, via email: [v.geskus@student.maastrichtuniversity.nl](mailto:v.geskus@student.maastrichtuniversity.nl)
