# Supplementary material for: Stakeholder perspectives on implementing geographic information systems for leprosy elimination in Pakistan: A qualitative study
Source: PLoS One. 2026 Nov 5;21(11):e0360428. doi: 10.1371/journal.pone.0360428 (PMC13739172; doi:10.1371/journal.pone.0360428)
Supplement: Supplementary file 3 [file pone.0360428.s003.docx]

## Appendix 3: Informed Consent

**Title of the Research**: Mapping the Neglected, Making them Visible; *Qualitative Study Investigating the Role of GIS Tools and Spatial Analysis to Tackle Leprosy in Pakistan*

**Institutions**: Marie Adelaide Leprosy Centre (MALC) Pakistan; University of Maastricht (UM)

**Principle Investigator:** Vincent Geskus, Student Health and Digital Transformation (UM)*;*
**Co-Investigators:** Anil Fastenau (MALC); Johan van Soest (UM); Matthew Willis (MALC)

**To be filled out by participant:**

Dear participant,

Thank you for being part in this study, investigating the challenges and opportunities for implementing Geographic Information Systems (GIS) to support active case findings for leprosy. By signing this form, you confirm that you have read and understood the information about the study as provided in the information sheet, and that any questions, doubts or concerns have been delt with fully and respectfully. The purpose of the study has been clearly explained to you, and you have been informed about potential risks associated with your participation. You agree that the interview will be audio recorded on an external recording device you are comfortable with your interview responses being analysed anonymously, with the possibilities of your answers being included in scientific publications. You consent to the storage of the interview data on digitally encrypted files for a period of 10 years following the final publication. You also agree that interview transcripts will be shared with MALC and UM but not with any external third party. You understand that participation to this study is entirely voluntarily, and you retain the right to withdraw at any time without providing a reason. This includes skipping any question or to stop the interview at any point you wish. You have been reassured that such decisions will not impact your personal or professional relationship with MALC or UM.

- I, ....................................... (name of participant), hereby give my consent to be a participant in this study

Date: .....................................................	Signature: ...................................

**To be filled out by the researcher:**

I declare that I have informed the participant to the best of my ability, providing all available information before the start of the interview and answering their questions truthfully. The participant has been made aware of the study purposes, procedures, risks and privacy concerns, to which the participant has agreed without any coercion. I pledge to uphold all agreements made with the participant regarding their consent to participate in the study throughout the entire research process.

Name of researcher: .....................................................................................................

Date: ................................	Signature: ...............................................
