# Supplementary material for: Stakeholder perspectives on implementing geographic information systems for leprosy elimination in Pakistan: A qualitative study
Source: PLoS One. 2026 Nov 5;21(11):e0360428. doi: 10.1371/journal.pone.0360428 (PMC13739172; doi:10.1371/journal.pone.0360428)
Supplement: Supplementary file 4 [file pone.0360428.s004.docx]

## Appendix 4: Extended Positionality Statement

When writing this master thesis, it is important to acknowledge my positionality and the place I take up in society, and to put this into perspective to the research I have conducted. I am aware that who I am might have impacted the way this study is designed, performed, and analysed. Although I always strive to conduct research that is as neutral and free from bias as possible, it goes without saying that who I am as a person does influence the way I work. To start with perhaps the most evident influence: my positionality impact the topics I choose to care, engage and write about.

Last year, within my Master of Global Health, I decided to conduct a political discourse analysis on Extinction Rebellion, as it is my personal believe that the climate crisis, and the lack of political will to take serious measures to fix generation of injustice, is THE biggest health crisis of our time. While in such a study I still upheld all scientific dogma’s, it, once again, went without saying that I had some personal bias within this research. For example, my personal opinions were expressed in which political views I found more harmful than others. Yet, these believes would always be supported by well-grounded arguments and literature, rather than just pure personal preferences

While the topic of my thesis this year is fundamentally different compared to last years, the decision was still grounded in personal believes and motivations: without reducing global disparities in health access and control, we cannot speak of true health equity. Albeit it be a bit of a selfish source of motivation, I like to have the feeling that I can make a personal contribution to bridging such health gaps. At the same time, I realize that I can make such contributions considering I take up a privileged position in this global society: I live in a rich country and I have access to high levels of education. These conditions allowed me to do research in a context, in this case Pakistan, where such circumstances are not broadly available. This stark contrast between my circumstances and those of the communities I study offers a valuable diversity of perspectives, but it also introduces ethical challenges. To create a bond of trust with you, the reader, I will in the upcoming paragraphs reflect on some of the main challenges I encountered, and how these might have impacted my study.

First of all, I am a young, white, cis-man, coming from a relatively privileged background, who has been living in the Netherlands for his whole life. I have never been in touch with someone who has leprosy. Leprosy is considered a past station where I live and has only really come to my attention when starting on this project. While aware of what for negative consequences leprosy can have, it is thus hard for me to imagine that this disease is still a public health issues in other places in the world. Given Pakistan’s status of a low-endemic country, to me it is evident that new and innovative methods are required to act as a final push in eliminating leprosy, which makes me convinces of the strength and added value of GIS. However, I did not base those beliefs on actual experiences from within the local context. Even though I talked with local professionals and read a lot of research, my beliefs were nonetheless developed and evolved from behind my desk. In my view, this is a serious limitation for any type of research, but especially for qualitative research.

My thesis last year made me realize the importance of going into the real world while performing research. I did this by going to multiple Extinction Rebellion protests, which was very important for myself to develop a full understanding of the concept I had been reading on. While I cannot say what I would have gained specifically by going to Pakistan, to me it is evident that this would have resulted in additional insights into the nuances within specific local contexts in the country. Now, at the end of the day, I am still just a student from Europe, that made a bunch of recommendations by spending hours upon hours behind his laptop: how would we know for sure that the most important points are captured? This does not take anything away from the work I have performed, as I know that I have worked in a dedicated and integer manner. However, this acknowledgment was required as it cannot be overlooked.

Perhaps one of the most paramount challenges I experienced within this thesis was the difficulty in overcoming language barriers. During my Global Health studies, I have gained hands-on experience with these types of online communication stretching cultural and linguistic boundaries. I learned that issues such as poor connectivity and quality of computer devices can seriously hinder effective communication. While in my thesis all interviews were conducted in English, it sometimes proved difficult to create a shared conceptual understanding of my question; a barrier that was reinforces through recurrent connectivity issues during the interviews. While trust building and patience helped me to minimize misunderstandings, it was not possible to avoid them completely. Additionally, while I felt a bond of trust, it is hard to tell if the participants felt the same. Yet, I can stress, at least from my position, that I have felt welcomed in every conversation I had during this research. Whether this was with the experts that often came from similar cultural backgrounds, or with the local Pakistani professionals, I always felt a good connection while having such conversations. The interviews reminded me of the strength of cross-cultural collaboration: there is nothing more beautiful and impressive than to cut through stigmas and prejudices, especially in times where our differences are amplified by harmful political rhetoric of exclusion by “othering” those who do not think alike.

Lastly, I feel like it is good to spent some extra words on the personal circumstances through with I wrote this thesis. For some years now I have been struggling with obsessive and compulsive behaviour which has put a serious strain on my mental health. For a long time, I have considered my behaviour almost as some sort of superpower, as something to be “romanticized”: my obsessiveness would allow me to almost behave like a brilliant detective (almost like a Sherlock Holmes), thereby getting better results and doing research with a very keen eye on small details (which would run through my head as endless thought spirals). I contributed this behaviour for example to my successes I booked at university, especially in comparison to high school: all of a sudden I got high grades, how amazing is that?! Luckily, I no longer see it that way, as it is really something that puts a strain on my functioning on a day to day basis. I tend to focus too much on details that are at best of little relevance, I tend to include way too much information out of a fear that something is missing, and I doubt every word I write down out of an irrational fear of plagiarism, which often results in me writing down sentences which are sub-optimal, just to cope with my obsessive thoughts. Even this extended positionality statement is to some degree an attempt to deal with some of my obsessive thought spirals! And these are only the ways it impacts me within writing the thesis, thus not including other behaviour in my day-to-day life. All with all, it can put a big strain on not only my time but also my energy to do the things that really matter to me in life.

For those wandering why I am including this reflection on myself, I will say the following: I wanted to include this in my positionality statement, not only to break down the stigmatization that can hang around mental issues like OCD, but also as I believe that can shine some extra light on how this research ended up the way it did. I think one of the clearest examples of how this impacted the study conducted can be found within the interview guide, which is very long and extensive, stemming from my fear of missing out on important aspects. Perhaps it would have been better to have a more dedicated focus on more specific parts of implementation. Others will probably praise the guide as very complete and insightful to the broad scala of implementation challenges of GIS in Pakistan. Nevertheless, this is how I did it, which is something deeply ingrained within who I am, and thereby thus impacting how this study turned out the way it did. The people that read this thesis with close attention will probably find more examples in which this comes back, and realize that my tendency to get stuck in obsessive thoughts can work both in my favour as against me.

Overall, the task I took on was a big challenge, on multiple levels. Being an outsider is not always a bad thing, as it also enabled me to look at this topic with fresh ideas and fewer preconceptions, which hopefully reflect well on the work I delivered. Nevertheless, it was a challenge that made me nervous and doubt myself from time to time, but ultimately I can say that I am glad that it brought me in contact with many fantastic people doing important work. My personal experiences are captured within this thesis, as you cannot see the researcher as a separate thing from his work. Nonetheless, I hope to have given you enough insight into who I am and what drives me, so that you and other readers can form your own opinion on how this influenced the research. If there are questions that remain, please do not hesitate to contact and reach out to me: I would always be happy to explain decisions that I have made! To make a complete end to this thesis, I would like to finish off by adding a quote from a recent book that I read by John Green (*Everything is Tuberculosis*), remembering us that any sickness, whether it is OCD, leprosy or anything else, is much more than just a medical concept.

*“And so we must remember that illness is not only a biomedical phenomenon, but also a constructed one, and how we imagine leprosy or OCD or tuberculosis matters.”*
